# Indents

**Published:** 1902-12-15

**Authors:** 


### INDENTS.

☞ **Brief Articles of Technical or Scientific Value will receive notice and comment. Contributions invited.**

Toothache Drops.—

| | | |
|---|---|---|
| ℞ | Menthol - - - | grs. 30 |
| | Camphor - - - - | grs. 15 |
| | Cocain - - - - | grs. 3 |

Mix and triturate to liquefaction and apply to carious cavity on cotton.

Receding Gums.—Apply glycerite of tannin, (made by dissolving one ounce of tannic acid in four ounces of glycerine by gentle heat), to spongy or debilitated gums as a tonic astringent lotion. It condenses soft gums and reestablishes the nutritive functions.

Bleaching Pink Rubber.—I have found that the quickest way to bleach pink rubber is to focus upon it the rays of the magnifying glass, taking care not to burn it, as this method gives you a beautifully bleached pink in about five minutes.—*L. Arndt, D.D.S.*

Removing Plaster.—Sugar placed in water, or the use of simple syrup, will greatly facilitate the removal of plaster of Paris from the hands after applying plaster dressings. The use of sweet oil is also serviceable for this purpose.—*Internat. Jour. of Surgery.*

Amalgam and General Health.—Dr. Morrison, of Peterboro, Canada, in the September *Cosmos*, reports two cases of immediate recovery of the patients from long illness, when the amalgam fillings had been removed from their teeth and gold and cement substituted.

Tin Foil on Vulcanite.—After the rubber is packed in one-half of flask, adapt No. 20 tin foil to the face of the cast, and cover it with a thin coating of soap to make it peel off the plate easily. This will give a good surface to the palatal surface when vulcanized.—L. P. Haskel, in *Brief*.

Joining the Dental Society.—Pertinent were the remarks of a member of a State society, relative to non-members in the State. He said: "If they know more than we, let them join, and teach us something; if they know less, let them join, and in turn be taught."—C. J. Grieves, in *Cosmos*.

For Spongy gums.—

℞. Acetate of lead..........grs x

Tincture of opium..........ʒ j

Rose water q. s. ad........℥ j

Apply as a lotion by means of a pledget of cotton two or three times each day. Shake the bottle well before using.—J. R. Megrow, D.D.S., in *Western Journal*.

Spittoons, to Keep Clean.—Mr. A. W. Wright, Jr., of London, finds that a little sulphate of copper sprinkled inside the spittoon (metal or earthenware), before the day's operations commence, prevents any unpleasant odor, does not allow the interior of the spittoon to become furred, and renders it much easier to cleanse than usual.—*Ash's Circular*.

To Reharden Steel Instruments.—To reharden tools that have been burned they should first be cleaned. Then take common machine oil and forge cinders ; make a bed of the cinders, heat the tools, put them in the cinder bed, and pour the oil on them till they are partly cooled off. Having put plenty of oil on the tools, cover them with the cinders and ashes, burying them deeply. They should be left so for twelve or fifteen hours.—*Power and Transmission.*

Immediate Root Filling.—It may be practicable and proper to fill the canal from which a living pulp has just been removed, providing the canal is or can be made sterile. But no canal which has contained a putrescent pulp should be filled at the same sitting at which it is opened. Its disinfection can only be positively accomplished by repeated disinfection with dressing of creosote, after all infected dentine that can be spared has been removed by instrumentation.—Dr. D. D. Smith, in *Digest.*

Removing a Pin from a Root.—When a pin is broken in a root and is held firmly by the cement, it can be removed by grasping the end with a pair of pliers and using a strong, narrow chisel as a lever between the end of the root and the pliers, care being taken not to chip off any frail edges of the root. The operation is much like prying out a tack with a screw-driver. It is often necesssary to remove the cement from the end of the pin far enough to insure a strong grasp with the pliers.—R. B. Tuller in *Review.*

Menstrual Neuralgia.—Approaching the menstrual periods, women are often afflicted with neuralgia from hyperemie conditions of the pulp or peridental structures. In such cases I have had good results follow the internal administration of one or two five grain tablets of ammonol every two hours. —J. R. Megrow, in *Western Journal*. We have had better results from giving two five grain tablets and following this, with one-half tablet every hour until three have been taken, then one-half every two hours if necessary.—*Ed*.

Hare Lip.—Ratchford reports a strange history of a family in which four girls were born with "hare lips" and "cleft palates"; but three boys in the same family have no trace of either deformity. A curious fact also in this connection is that the mother in every case predicted the sex of the child before it was born, and also whether it would be deformed or not. There was a family history of tuberculosis on both sides but no history of cleft palate. The mother had a high arch. The query is; could any mental impression on the mother have caused the "hare lip".—*Arch. of Pedriatrics*.

Implanting Artificial Roots.—Dr. J. L. Williams, of London, Eng., places in the sockets, of irredeemable roots, after deepening the sockets, a conical-shaped screw (made after the pattern of a root, spirally cut) of platinum, hollow I believe, it may be of tin or lead. He made his deductions of success in these cases (of retention by nature of such foreign metals) from the fact that metallic balls have been retained in the body in many cases without discomfort. The screw is permitted to rest in the socket for some three or four weeks when it is unscrewed, a porcelain crown attached and then replaced.—*Review*.

Menthol Preliminary to Anesthesia.—Mentholization of the mucosa of the air-passages before, during, and after etherization, has given Dr. W. A. Briggs (*Amer. Med.*, April 26, 1902) such satisfaction as to impel him to submit the method to the profession at large. The method is as follows: Sprinkle a fluid drachm of oil of peppermint or of saturated alcohol solution of menthol in the cone; let the patient inhale it freely for three minutes, then saturate the cone with ether and bring it down slowly over the face; after a few full inhalations crowd the cone down well and push the etherization as rapidly as is consistent with safety; continue the use of the mentholized cone through the whole period of anesthesia, replenishing the ether as usual. After the operation, let the patient inhale oil of peppermint or menthol from a handkerchief, freely, and often until the tendency to nausea subsides.—*Merck's Report.*

Electricity as an Anesthetic.—A method of replacing the ordinary anesthetics used in dental surgery by the action of high-frequency currents has been brought out by Messrs. Regnier and Didsbury, of Paris (*Sci. Amer.*, LXXXVII, p. 71). d'Arsonval had already shown that high-tension and high-frequency currents have a local anesthetic effect, and the experimenters wished to see whether this could not be used to advantage for dental operations, and so do away with the inhalations of gas, which are not without danger to the patient. In the case of extraction they found it to work quite successfully. The apparatus they employed afforded a current which gave the patient no sensation other than a slight heating in the region covered by the electrodes. It was found that a tooth with one root was made completely insensible by the application of a current of 150 milliamperes for 3 to 5 minutes, while the larger teeth needed 200 to 250 milliamperes for 6 to 8 minutes. As to the use of the method for more prolonged operations, the experiments are not yet conclusive, although they are favorable on the whole.—*Merck's Report.*

New Cure For Pyorrhoea Alveolaris.—Dr. Fingen, of Copenhagen (the gentleman who has gained such reputation in the cure of lupus vulgaris, a tubercular condition, by the use of the arc light, the rays of which are concentrated by means of lenses) discovered that patients who came under his care while suffering from other diseases of the face but who were also affected with pyorrhoea, some whose teeth were so loose that they seemed beyond hope, after undergoing a course of arc light treatment would, together with their other cure, find their teeth had become absolutely firm in their sockets. Dr. Fingen followed such cases and found that the cure was lasting. Dr. W. W. Walker, of New York, was so impressed with what he saw that he promises to install such an outfit as Dr. Fingen uses and is certain of success. He will also keep the society informed of the progress of the work.

The principle of the arc light cures is the destruction of germs which it is claimed respond very readily to its action. No cleansing of the teeth is done by the dentist at any time during the course.

The treatment is applied from one-half to three-quarters of an hour for twenty-one days, then a rest of twenty-one days is allowed and so continued for six months.—*Review.*

